# Prevalence of initial orthostatic hypotension in geriatric inpatients: the role of time to stand

**DOI:** 10.1007/s41999-025-01339-0

**Published:** 2025-10-30

**Authors:** Ahmed N. Hassona, Tania Zieschang, Nadine Poelker, Johanna Michel, Tim Stuckenschneider

**Affiliations:** Department for Health Services Research, Geriatric Medicine, School of Medicine and Health Services, Carl von Ossietzky University, Ammerländer Heerstraße 114-118, 26129 Oldenburg, Lower Saxony Germany

**Keywords:** Initial orthostatic hypotension, Orthostatic hypotension, Blood pressure regulation, Geriatric inpatients, Frailty, Continuous blood pressure monitoring

## Abstract

**Purpose:**

Initial orthostatic hypotension (IOH) is a clinically relevant blood pressure abnormality that remains underrecognised in older adults, particularly in hospitalised or frail populations. This study aimed to determine the prevalence of IOH in geriatric inpatients, compare clinical characteristics between individuals with and without IOH, and assess the diagnostic value of different measurement intervals.

**Methods:**

Continuous beat-to-beat blood pressure monitoring was used in geriatric inpatients. The test protocol included 5 min supine, 3 min standing, and 2 min recovery supine. Two diagnostic time windows were analysed: (1) within 15 s of the standing command, and (2) within 15 s after a stable upright posture. Clinical and demographic characteristics were compared between participants with and without IOH.

**Results:**

IOH was identified in 31.4% out of 137 patients (64% female; mean age 84 ± 6 years) when both diagnostic windows were considered. No statistically significant differences in clinical or demographic characteristics were observed between groups, although some non-significant trends, such as slightly longer hospital stay in the IOH group, were noted. The most pronounced blood pressure declines occurred within the first 30 s of the standing command (systolic: *p* = 0.005; diastolic: *p* < 0.001). The median time to achieve postural stability was 26.6 s.

**Conclusion:**

IOH is common in geriatric inpatients, but often goes undetected due to its transient nature and delayed postural stabilisation, which affects diagnostic accuracy of traditional techniques. Continuous monitoring starting at the postural transition improves detection and may inform targeted interventions, particularly in high-risk patients, to reduce complications and optimise recovery.

## Key summary points

**Aim:**

To determine the prevalence of initial orthostatic hypotension (IOH) in hospitalised older adults, compare the clinical characteristics between those with and without IOH, and evaluate the diagnostic value of different measurement intervals.

**Findings:**

IOH was present in 31.4% of geriatric inpatients. The greatest blood pressure drop occurred within the first 30 s after standing up, and patients with IOH exhibited a non-significant trend towards longer hospital stays.

**Message:**

IOH is common, but frequently overlooked in geriatric inpatients; continuous blood pressure monitoring enhances detection by covering transition times and, thus, may inform targeted interventions.

## Introduction

Initial orthostatic hypotension (IOH) is a clinical syndrome characterised by a rapid decline in blood pressure immediately following active standing. It is defined as a decrease in systolic blood pressure (SBP) of ≥ 40 mmHg and/or diastolic blood pressure (DBP) of ≥ 20 mmHg within the first 15 s of standing, often accompanied by symptoms of cerebral hypoperfusion, such as lightheadedness or dizziness [1, 2]. Its immediate nature distinguishes it from classical orthostatic hypotension (COH), which is characterised by a sustained reduction in SBP of at least 20 mmHg or DBP of at least 10 mmHg within 3 min of standing or head-up tilt to at least 60° on a tilt table [1]. However, recent research has proposed that IOH should be considered a distinct condition, warranting independent reporting even in individuals who also meet the diagnostic criteria for classical OH, as the co-occurrence of both conditions reflects a distinct autonomic and haemodynamic response with potential clinical implications [3].

Despite its clinical relevance, IOH remains frequently overlooked in clinical practice, particularly among older adults [2]. Studies indicate that its prevalence reaches 27.8% in community-dwelling older adults, 35.2% among geriatric outpatients, and 10.0% in institutionalised individuals, with incidence increasing due to age-related autonomic dysfunction, polypharmacy, and comorbidities [4]. IOH has been linked to adverse outcomes, including falls, syncope, frailty, hospitalisations, and increased mortality in older adults. Thus, its recognition is crucial for preventing injuries and improving patient outcomes, which underscores the urgent need for accurate assessment methods [5–7].

The duration of the transition from sitting to standing plays a critical role in haemodynamic stability during the initial seconds of upright posture, particularly in older adults, as highlighted by van Twist et al. in their narrative review [8]. Among the few studies that specifically examined the speed of standing up, O’Connor et al. and De Bruïne et al. found that slower transitions were associated with attenuated orthostatic blood pressure declines [9, 10]. In both studies, standing-up times varied substantially -from approximately 5 to over 20 s - despite participants being community-dwelling older adults. However, data on standing-up speed and its haemodynamic effects in institutionalised or highly frail older individuals remain scarce. Therefore, further research is warranted to assess the impact of standing-up speed in highly vulnerable populations, such as geriatric inpatients, where frailty often prolongs the sit-to-stand transition. A slower ascent may facilitate blood pressure stabilisation by enhancing venous return through improved muscle pump activity, thereby mitigating orthostatic blood pooling [11]. However, given that geriatric patients frequently exhibit impaired compensatory mechanisms due to sarcopaenia, reduced vascular compliance, and diminished autonomic reflexes, the potential stabilising effect of prolonged standing-up times remains unclear and requires further investigation [12].

Assessing IOH in vulnerable populations requires highly sensitive measurement techniques. Conventional intermittent blood pressure measurements often fail to capture the rapid and transient blood pressure fluctuations associated with IOH, potentially leading to underdiagnosis [13–15]. In contrast, continuous, non-invasive beat-to-beat blood pressure monitoring offers a more precise and comprehensive assessment, enabling real-time detection of blood pressure changes and providing critical insights into cardiovascular control mechanisms during postural transitions [16].

This study aims to determine the prevalence of IOH in geriatric inpatients, compare the clinical characteristics of patients with and without IOH, and evaluate different time frames to identify the most effective detection strategies, particularly for high-risk populations.

## Methods

### Study design

This cross-sectional study was conducted at Klinikum Oldenburg (KOL), a 625-bed tertiary care centre in northwestern Germany, which serves as a teaching hospital affiliated with the Carl von Ossietzky University Oldenburg (CVO). The study focused on two geriatric wards comprising a total of 35 beds: the University Clinic for Geriatrics and the Geriatric Trauma Centre (ATZ), jointly managed by the University Clinics for Orthopaedics, Trauma Surgery and Geriatrics. Care was provided in accordance with the German OPS 8–550 framework for acute geriatric early complex treatment. Among other obligatory requirements concerning the multiprofessional team, assessments, and team conferences, the OPS 8–550 also specifies minimum treatment durations (8–550.0/0.1/0.2: ≥ 7/ ≥ 14/ ≥ 21 treatment days with ≥ 10/ ≥ 20/ ≥ 30 units of ~ 30 min, predominantly individual therapy). These requirements structurally prolong length of stay compared with short-stay acute wards [17, 18] and geriatric acute care in other countries. Ethical approval was obtained from the Medical Ethics Committee at CVO (approval number 2023–112), and the study adhered to the principles of the Declaration of Helsinki. Additionally, the study was prospectively registered with the German Clinical Trial Register (Deutsches Register für klinische Studien, DRKS00031905).

### Participants

A consecutive sampling approach was used to achieve the targeted sample size. Participant screening was conducted at irregular intervals during the day shift between June 2023 and June 2024. Eligibility for participation required admission to the geriatric ward or the “Geriatric Trauma Centre” at the Klinikum Oldenburg, the ability to stand with support for at least 3 min, and medical clearance to perform the study protocol. Participants were also required to demonstrate the capacity to provide written and dated informed consent. Individuals were excluded if they exhibited acute delirium, as determined by clinical assessment.

Potential participants were identified during medical rounds by a physician, who then informed the study team about eligible individuals. A medical doctor or a member of the study team subsequently approached these individuals to explain the purpose and procedures of the study. Sufficient time was provided to address any questions, and participants were given the opportunity to reflect on their decision for 24 h. Measurements were initiated only after written informed consent had been obtained.

For participants with limited capacity to consent, such as those with severe dementia, the study followed ethical guidelines for research involving adults who lack decision-making capacity. This included adherence to the principles of group benefit, subsidiarity, and minimal risk [19]. In such cases, consent forms were signed jointly by the participant and, when necessary, a legal guardian or family member, who was consulted in collaboration with the participant. To enhance understanding, study materials were adapted to meet the needs of individuals with cognitive impairments and provided as required.

### Data collection

Before measurements, participants were interviewed to obtain demographic and clinical characteristics (age, sex, height, weight), history of falls in the past 12 months, presence and frequency of peripheral oedema, and use of compression stockings. Following the interview, blood pressure was measured with the Finapres NOVA, a validated system for non-invasive, continuous beat-to-beat monitoring via finger cuff. Study personnel received standardised training in device operation. Participants rested supine for 10 min to establish baseline values, followed by 5 min of continuous supine monitoring. They were then instructed to stand up, and blood pressure and heart rate (HR) were continuously recorded for 3 min in the upright position. The procedure concluded with an additional 2 min supine rest period, following standard protocols to assess any orthostatic dysregulation [16]. Outcome parameters included SBP, DBP, and HR, all obtained using the Finapres NOVA system. A flowchart or timeline of the OH assessment protocol can be found in Finucane et al., with our dual-timing modification representing an additional methodological step. The presence of orthostatic intolerance symptoms was assessed immediately after blood pressure measurement. Participants were asked whether they experienced dizziness, blurred vision, faintness, lightheadedness, or headache during or immediately after standing up. To minimise bias, no symptom screening was performed before testing, and examiners were blinded to potential symptom history.

Data from interviews were cross-checked with electronic health records to validate self-reported weight and height. Functional status (Barthel Index), length of hospital stay, and admission diagnoses were extracted from electronic records. Diagnoses were coded according to the International Statistical Classification of Diseases and Related Health Problems, 10th Revision, German Modification (ICD-10-GM; 2023/2024) and grouped a priori into clinically coherent categories (e.g. cardiovascular and cerebrovascular disorders; injuries and fractures; infectious and parasitic diseases; syncope/dizziness; pneumonia; and other less frequent diagnoses (e.g. digestive system disorders, endocrine, nutritional, and metabolic diseases, COPD, neuropsychiatric disorders, cancer, dyspnoea, orthostatic hypotension) [20]. Medical comorbidities, current medications, and associated clinical information were extracted from patients’ medical records. Collected variables included cardiovascular disease, diabetes mellitus, Parkinson’s disease or dementia; the total number of medications prescribed, and the use of specific drug classes such as diuretics, beta-blockers, ACE inhibitors or angiotensin II receptor blockers (AT1-blockers), calcium channel blockers, alpha-blockers, antidepressants, benzodiazepines, and antipsychotics.

### Blood pressure data analysis

Beat-to-beat blood pressure and HR data were exported to MATLAB (Version R2022b; MathWorks, Natick, MA, USA) and processed using a 5-s moving average filter to minimise artefacts. This averaging interval was selected based on previous findings indicating its strongest association with OH and fall history compared to beat-to-beat and other time-averaging methods [21]. Given the continuous nature of beat-to-beat measurements, the moving average was applied to five consecutive values rather than a fixed time duration. To ensure data reliability, measurements with poor signal quality, characterised by excessive fluctuations or artifacts, were excluded from the final analysis. In cases of brief signal loss, linear interpolation was employed to fill data gaps, following established methodologies.

The assessment of IOH was based on supine SBP and DBP, defined as the mean blood pressure recorded during the final 60 s prior to standing up. The relative decrease in blood pressure was determined by subtracting the lowest recorded value within four predefined time intervals from the supine measurement. These intervals included 15 and 30 s after the command to stand up, as well as 15 and 30 s after achieving an upright standing position. HR was analysed using the same time intervals with HR changes calculated by subtracting the supine HR from the mean HR recorded after standing up at each respective interval.

IOH was defined as a decrease of ≥ 40 mmHg in SBP and/or ≥ 20 mmHg in DBP within 15 s of active standing, whereas classical OH was defined as a sustained decrease of ≥ 20 mmHg in SBP and/or ≥ 10 mmHg in DBP within 3 min of active standing [1]. For comparative analyses, individuals who met the criteria for IOH in either the interval following the command to stand up or after reaching the upright position were classified as having IOH.

### Statistical analysis

All statistical analyses were performed using IBM SPSS Statistics 29 (IBM Corp., Armonk, NY, USA). Categorical variables were presented as absolute frequencies (n) with percentages (%), while continuous variables were summarised as means with standard deviations (SDs) for normally distributed data or as medians with interquartile ranges (IQRs) for non-normally distributed data. The Shapiro–Wilk test was used to assess data normality.

Comparisons between groups stratified by diagnosis were performed using one-way ANOVA for normally distributed continuous variables or the Kruskal–Wallis test for non-normally distributed continuous variables. For categorical variables, comparisons were performed using Chi-square (χ^2^) tests. If a statistically significant difference was identified in the overall comparison (*p* < 0.05), post hoc pairwise comparisons were performed using Bonferroni correction to adjust for multiple testing, with adjusted significance levels clearly reported.

Within-subject comparisons of blood pressure and HR measurements at different time points (e.g. immediately after the command to stand up and after achieving a stable upright posture) were performed using the Wilcoxon signed-rank test, as the data were not normally distributed according to the Shapiro–Wilk test. Statistical significance was set at *p* < 0.05 for all analyses. Figures were generated in MATLAB R2022b.

## Results

### Patient characteristics

**Fig. 1:**
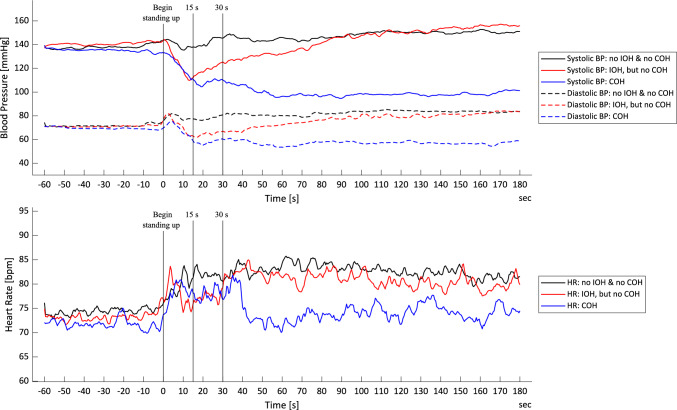
Haemodynamic responses to active standing stratified by orthostatic hypotension status. SBP, DBP, and HR responses to active standing are shown for three groups: no IOH and no COH (black lines), IOH but no COH (red lines), and COH (blue lines). Vertical lines indicate the beginning of standing (0 s) and critical intervals at 15 s and 30 s post-standing. Continuous lines represent systolic values, anddashed lines represent diastolic values. All data were filtered using a 5-s moving average filter; IOH = present if diagnosed based on 15/30 s after standing up or 15/30 s after stand marker

A total of 149 geriatric inpatients were initially considered for this study. Twelve patients were excluded from the final analysis due to excessive signal fluctuations or artefacts, resulting in a study cohort of 137 patients. Among these, 54 patients (39.4%) had no OH, 43 patients (31.4%) met the criteria for IOH, and 40 patients (29.2%) had COH, including 33 patients who also fulfilled the criteria for IOH. The spectrum of haemodynamic responses to active standing across groups categorised by orthostatic hypotension status is shown in Fig. 1.

No significant differences were found between groups regarding age, sex, BMI, Barthel Index, comorbid cardiovascular disease, diabetes mellitus, dementia, Parkinson’s disease, number of medications, or baseline supine systolic and diastolic blood pressure (*p* > 0.05). Also, the prevalence of oedema, previous falls, and orthostatic intolerance symptoms did not differ significantly between groups. Among the participants, the most frequent admission categories were cardiovascular and cerebrovascular disorders (*n* = 36), injuries and fractures (*n* = 23), infectious and parasitic diseases (*n* = 13), syncope/dizziness (*n* = 11), and pneumonia (*n* = 10). Less frequent categories included digestive system disorders, endocrine, nutritional, and metabolic diseases, COPD, neuropsychiatric disorders, cancer, dyspnoea (*n* < 10), and orthostatic hypotension (*n* = 2). No differences between diagnoses existed among the subgroups (*p* = 0.504).

The use of compression stockings differed significantly across groups (*p* = 0.033). However, after Bonferroni correction for multiple comparisons (adjusted *α* = 0.017), none of the pairwise group differences remained statistically significant. The smallest p-value was observed between the COH group and the group without OH (corrected *p* = 0.057). Although individuals with IOH showed a tendency towards longer hospital stays, this difference did not reach statistical significance (*p* = 0.058) and remained non-significant across all pairwise comparisons. Patient characteristics stratified by OH status are presented in Table 1.

**Table 1:** Patient characteristics for the full sample, stratified by orthostatic hypotension status

	No OH (*n* = 54)	IOH (*n* = 43)	COH (*n* = 40)	*p*-value
Age, years	83.2 ± 6.2	85.1 ± 5.7	84.1 ± 6.7	0.340
Sex, n (male(m)/female(f))	19 m/35f	16 m/27f	15 m/25f	0.967
BMI (kg/m^2^)	27.2 ± 6.3	25.1 ± 5.6	24.6 ± 4.4	0.061
Medical history and medication				
Comorbid diseases				
Cardiovascular disease, yes, n (%)	49 (90.7)	40 (93.0)	37 (92.5)	0.909
Diabetes mellitus, yes, n (%)	10 (18.5)	10 (23.3)	12 (30.0)	0.429
Parkinson’s disease, yes, n (%)	0	0	0	-
Dementia, yes, n (%)	4 (7.4)	3 (7.0)	2 (5.0)	0.890
Medications number	7.7 ± 2.9	8.1 ± 3.5	9.1 ± 3.2	0.185
Diuretics, n (%)	28 (51.9)	17 (39.5)	22 (55.0)	0.318
Beta-blockers, n (%)	28 (51.9)	23 (53.5)	28 (70.0)	0.170
ACE inhibitor/AT1-blockers, n (%)	36 (66.7)	20 (46.5)	21 (52.5)	0.119
Calcium channel blockers, n (%)	15 (27.8)	6 (14.0)	8 (20.0)	0.248
Alpha-blockers, n (%)	5 (9.3)	7 (16.3)	7 (17.5)	0.447
Antidepressants, n (%)	5 (9.3)	5 (11.6)	9 (22.5)	0.162
Benzodiazepine, n (%)	1 (1.9)	1 (2.3)	0 (0.0)	0.646
Antipsychotics, n (%)	1 (1.9)	4 (9.3)	4 (10.0)	0.197
Barthel Index (total score)	63.8 ± 16.1	60.2 ± 14.0	58.4 ± 18.6	0.209
Supine blood pressure (mmHg)				
Systolic	134.9 ± 21.1	138.7 ± 22.4	137.7 ± 24.8	0.783
Diastolic	70.6 ± 11.8	70.6 ± 9.5	70.2 ± 13.8	0.971
Symptoms of orthostatic intolerance, n (%)	15 (27.8)	18 (41.9)	21 (52.5)	0.079
Length of stay (days)	16.8 ± 4.6	19.0 ± 6.3	18.2 ± 5.5	0.058
Compression stockings (no/yes)	42/12	33/10	22/18	**0.033**
Oedema				
Never	14	12	10	0.956
Rarely	6	6	5	
Occasionally	5	3	3	
Frequently	0	1	0	
Persistently	28	21	21	
Fall in the past 12 months (no/yes)	22/32	16/27	10/30	0.268

All variables are presented as mean ± SD unless otherwise indicated

IOH was defined as a decrease of ≥ 40 mmHg SBP and/or ≥ 20 mmHg DBP within the first 15 s of standing, while OH was defined as a sustained decrease of ≥ 20 mmHg SBP and/or ≥ 10 mmHg DBP within 3 min of standing. The Barthel Index measures functional independence, with higher scores indicating greater autonomy. Length of stay refers to the total duration of hospitalisation (in days). Compression stocking use was recorded as either used (yes) or not used (no). Oedema categories represent the frequency of fluid retention symptoms; no OH: individuals without IOH and without COH; IOH: individuals with IOH, irrespective of the measurement timing (either after command to stand up or after stable standing), but without COH; COH: individuals with COH, including 33 patients who also had IOH

*BMI*  Body mass index

*P*-values < 0.05 are considered statistically significant and are highlighted in **bold**

### Characteristics stratified by orthostatic hypotension subtype

Table 2 summarises patient characteristics stratified by OH subtype at two time points: (1) immediately following the command to stand up, and (2) after achieving stable standing.

**Table 2:** Patient characteristics stratified by initial orthostatic hypotension subtype with the time interval measured after the command to stand up versus after achieving a stable standing position

	No OH	IOH transient	IOH sustained	COH	*p*-value
After command to stand up	(*n* = 64)	(*n* = 31)	(*n* = 19)	(*n* = 23)	
Age, mean ± SD, years	83.1 ± 6.3	85.8 ± 5.4	84.9 ± 4.4	83.5 ± 7.9	0.321
Sex, n (male(m)/female(f))	24 m/40f	10 m/21f	7 m/12f	9 m/14f	0.953
BMI (kg/m^2^), mean ± SD	26.9 ± 6.1	25.1 ± 5.9	25.3 ± 4.9	23.9 ± 3.7	0.157
Medical history and medication					
Comorbid diseases					
Cardiovascular disease, yes, n (%)	58 (90.6)	29 (93.5)	19 (100)	20 (87.0)	0.440
Diabetes mellitus, yes, n (%)	12 (18.8)	7 (22.6)	8 (42.1)	5 (21.7)	0.209
Parkinson’s disease, yes, n (%)	0	0	0	0	-
Dementia, yes, n (%)	5 (7.8)	2 (6.5)	2 (10.5)	0	0.520
Medications number, mean ± SD	7.7 ± 2.9	8.3 ± 3.7	8.8 ± 3.2	9.4 ± 3.2	0.230
Diuretics, n (%)	30 (46.9)	15 (48.4)	11 (57.9)	11 (47.8)	0.865
Beta-blockers, n (%)	32 (50.0)	17 (54.8)	14 (73.7)	16 (69.6)	0.174
ACE inhibitor/AT1-blockers, n (%)	38 (59.4)	18 (58.1)	12 (63.2)	9 (39.1)	0.334
Calcium channel blockers, n (%)	17 (26.6)	4 (12.9)	1 (5.3)	7 (30.4)	0.092
Alpha-blockers, n (%)	7 (10.9)	4 (12.9)	4 (21.1)	4 (17.4)	0.672
Antidepressants, n (%)	6 (9.4)	4 (12.9)	3 (15.8)	6 (26.1)	0.257
Benzodiazepine, n (%)	1 (1.6)	1 (3.2)	0 (0.0)	0 (0.0)	0.729
Antipsychotics, n (%)	1 (1.6)	4 (12.9)	3 (15.8)	1 (4.3)	0.059
Barthel Index (total score), mean ± SD	62.5 ± 15.7	61.8 ± 14.8	60.8 ± 20.7	56.4 ± 15.9	0.497
Supine blood pressure (mmHg), mean ± SD					
Systolic	134.4 ± 20.6	140.1 ± 23.8	140.6 ± 31.7	136.5 ± 16.8	0.763
Diastolic	70.7 ± 11.7	70.3 ± 9.3	69.5 ± 15.2	70.9 ± 12.2	0.933
Symptoms of orthostatic intolerance, n (%)	19 (29.7)	13 (41.9)	9 (47.4)	13 (56.5)	0.147
Length of stay (days), mean ± SD	17.1 ± 5.2	19.5 ± 5.8	18.7 ± 5.5	17.4 ± 5.6	**0.034**
Compression stockings (no/yes)	51/13	23/8	11/8	12/11	**0.046**
Oedema					
Never	19	5	3	9	0.481
Rarely	7	5	3	2	
Occasionally	5	3	3	0	
Frequently	0	1	0	0	
Persistently	32	17	10	11	
Fall in the past 12 months (no/yes)	26/38	12/19	6/13	4/19	0.230

After stable standing	No OH (*n* = 72)	IOH transient (*n* = 23)	IOH sustained (*n* = 32)	COH (*n* = 10)	*p*-value
Age, mean ± SD, years	83.9 ± 6.3	84.8 ± 5.5	84.3 ± 5.8	83.6 ± 8.6	0.745
Sex, n (male(m)/female(f))	25 m/47f	9 m/14f	12 m/20f	4 m/6f	0.972
BMI (kg/m^2^), mean ± SD	26.5 ± 6.1	25.5 ± 6.2	24.7 ± 4.3	24.1 ± 4.4	0.339
Medical history and medication					
Comorbid diseases					
Cardiovascular disease, yes, n (%)	66 (91.7)	21 (91.3)	30 (93.8)	9 (90.0)	0.976
Diabetes mellitus, yes, n (%)	13 (18.1)	6 (26.1)	9 (28.1)	4 (40.0)	0.365
Parkinson’s disease, yes, n (%)	0	0	0	0	-
Dementia, yes, n (%)	5 (6.9)	2 (8.7)	2 (6.3)	0	0.827
Medications number, mean ± SD	7.8 ± 3.3	8.0 ± 3.2	8.6 ± 3.0	10.7 ± 3.1	0.085
Diuretics, n (%)	36 (50.0)	9 (39.1)	17 (53.1)	5 (50.0)	0.766
Beta-blockers, n (%)	39 (54.2)	10 (43.5)	21 (65.6)	9 (90.0)	0.061
ACE inhibitor/AT1-blockers, n (%)	46 (63.9)	10 (43.5)	19 (59.4)	2 (20.0)	**0.034**
Calcium channel blockers, n (%)	18 (25.0)	3 (13.0)	5 (15.6)	3 (30.0)	0.457
Alpha-blockers, n (%)	6 (8.3)	5 (21.7)	5 (15.6)	3 (30.0)	0.151
Antidepressants, n (%)	6 (8.3)	4 (17.4)	8 (25.0)	1 (10.0)	0.137
Benzodiazepine, n (%)	2 (2.8)	0 (0.0)	0 (0.0)	0 (0.0)	0.608
Antipsychotics, n (%)	4 (5.6)	1 (4.3)	4 (12.5)	0 (0.0)	0.417
Barthel Index (total score), mean ± SD	63.1 ± 15.2	59.8 ± 15.8	58.3 ± 19.1	58.5 ± 15.5	0.434
Supine Blood pressure (mmHg), mean ± SD					
Systolic	136.3 ± 22.2	136.2 ± 20.7	138.6 ± 26.8	137.7 ± 16.4	0.971
Diastolic	70.8 ± 11.2	69.9 ± 10.4	69.8 ± 13.7	71.7 ± 13.4	0.903
Symptoms of orthostatic intolerance, n (%)	27 (37.5)	5 (21.7)	16 (50.0)	6 (60.0)	0.296
Length of stay (days), mean ± SD	17.6 ± 5.6	18.8 ± 5.3	18.4 ± 4.9	16.9 ± 7.2	0.164
Compression stockings (no/yes)	55/17	19/4	15/17	8/2	**0.008**
Oedema					
Never	18	6	4	8	0.851
Rarely	8	4	1	4	
Occasionally	6	2	1	2	
Frequently	0	1	0	0	
Persistently	39	10	4	17	
Fall in the past 12 months (no/yes)	30/42	8/15	7/25	3/7	0.268

"After command to stand up" refers to values measured immediately following the instruction to stand. "After stable standing" refers to values measured once a stable upright position was achieved. Continuous variables are presented as means unless otherwise indicated. Categorical variables are shown as absolute numbers (n) and percentages (%). p-values reflect overall group comparisons using one-way ANOVA or Kruskal–Wallis tests for continuous variables and the Chi-square test for categorical variables. For variables with significant overall differences (*p* < 0.05), Bonferroni-corrected post hoc pairwise comparisons were performed. Statistically significant results are shown in **bold**. No OH—no initial orthostatic hypotension (IOH) and no classical orthostatic hypotension (COH); IOH transient—IOH present; COH absent IOH sustained—IOH present with coexisting COH; COH—COH present, IOH absent

When defining OH based on the measurements according to the intervals immediately following the command to stand up, significant group differences were observed in the use of compression stockings (*p* = 0.046) and length of hospital stay (*p* = 0.034). However, after Bonferroni correction, no statistically significant differences were found between individual groups (all adjusted *p*-values > 0.008).

After stable standing, significant group differences were observed for the use of ACE inhibitor/AT1-blocker (*p* = 0.034) and compression stockings (*p* = 0.008). After Bonferroni correction for multiple comparisons (adjusted *α* = 0.008), only the difference in compression stocking use between the "No OH" and "IOH sustained" groups (*p* = 0.003), as well as between the "IOH transient" and IOH sustained" groups (*p* = 0.007), remained statistically significant. Additionally, the pairwise comparison for ACE inhibitor/AT1-blocker use between the "No OH" and "COH" groups reached the threshold for significance (*p* = 0.008). However, all other comparisons were not significant after adjustment (adjusted *p* > 0.008). No further significant differences were observed for other patient characteristics across groups at either time point. All detailed results are presented in Table 2.

### Time to achieve standing position

**Fig. 2:**
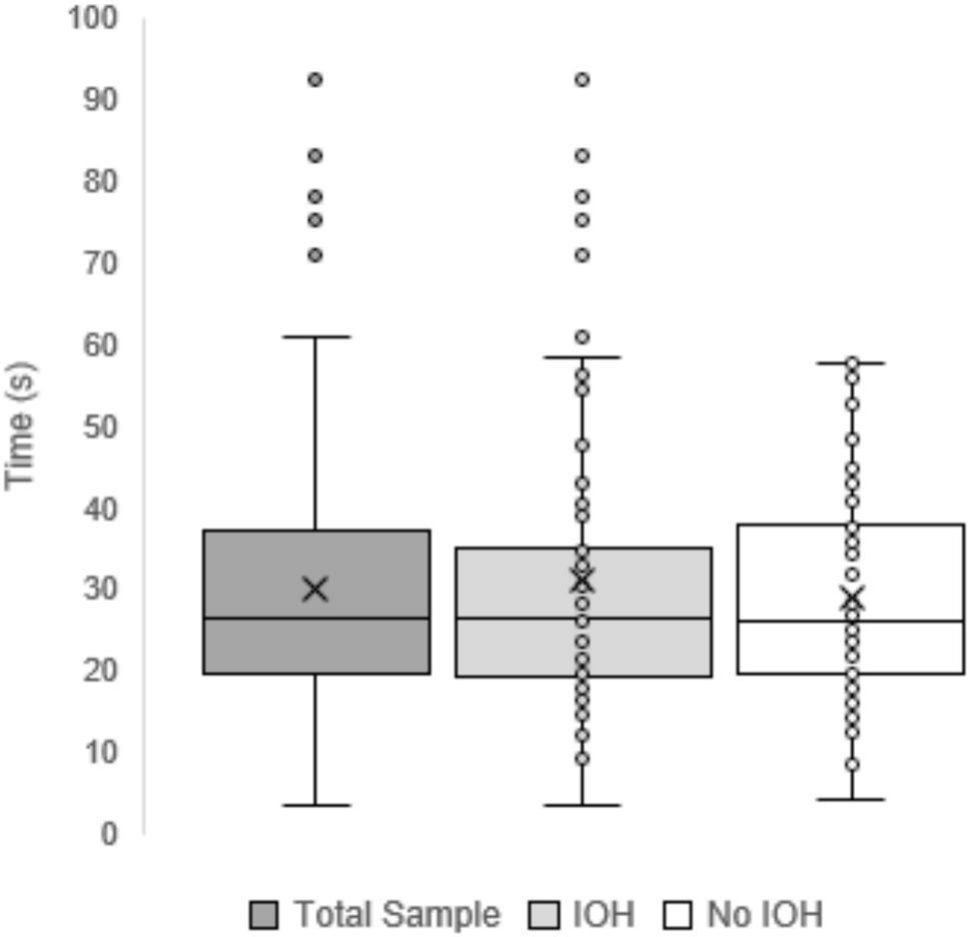
Time to achieve a stable standing position among geriatric inpatients

Figure 2 illustrates the distribution of the time required for participants to achieve a stable standing position. The median time to stand up for the overall sample was 26.4 s, ranging from 3.4 to 92.6 s. There was no significant difference in the median time to achieve a stable standing position between individuals with IOH (26.6 s, IQR: 16.0) and those without (26.2 s, IQR: 18.1) (*U* = 2328.5, *p* = 0.991). However, variability was greater in the IOH group.

### Blood pressure drops and HR changes relative to time to achieve standing

Blood pressure changes were assessed at 15 s and 30 sd intervals under two conditions: the command to stand up and after achieving a stable standing position. No significant differences were observed in the 15 s interval between the two conditions (systolic: *p* = 0.308; diastolic: *p* = 0.507). However, in the 30 s interval, blood pressure drops were significantly greater immediately following the command to stand up compared to the corresponding time frame after achieving a stable standing position (systolic: *p* = 0.005; diastolic: *p* < 0.001). Regarding HR variability, a statistically significant difference was observed within the initial 15 s interval with the increase in HR being less pronounced from the command to the time after standing (*p* = 0.046). Conversely, during the subsequent 30 s interval, HR variations did not reach statistical significance (*p* = 0.207) (Table 3).

**Table 3:** Comparative dynamics of blood pressure and HR responses within 15 and 30 s of standing

	Time since command (15 s)	Time after standing (15 s)	*p*-value	Time since command (30 s)	Time after standing (30 s)	*p*-value
SBP (mmHg)	29.2 ± 21.8	25.9 ± 30.5	0.308	38.3 ± 25.8	30.8 ± 31.7	**0.005**
DBP (mmHg)	11.4 ± 11.2	10.8 ± 20.6	0.507	30.8 ± 14.7	12.8 ± 21.9	** < 0.001**
HR difference (bpm), median (IQR)	5.63 (10.06)	8.51 (8.37)	**0.046**	7.39 (9.03)	8.45 (8.46)	0.207

Comparisons of blood pressure (SBP and DBP) and HR differences between paired measurements (time since command vs. time after standing) were conducted using the Wilcoxon signed-rank test. HR difference refers to the median change in beats per minute (bpm) between the supine position and postural transition. The specified intervals represent measurements recorded either immediately after the command to stand up or after achieving a stable standing position

*p*-values < 0.05 are considered statistically significant and are presented in **bold**

## Discussion

This study provides important insights into the prevalence and clinical significance of IOH among geriatric inpatients. A high prevalence of IOH (31.4%) was identified, emphasising that initial orthostatic dysregulation is a common condition in this population. While patients with IOH demonstrated a tendency towards prolonged hospitalisation, this difference was not statistically significant (*p* = 0.058) and remained non-significant across all pairwise group comparisons. Nevertheless, pronounced declines in blood pressure upon standing may contribute to delayed recovery or reflect increased physiological vulnerability. Beat-to-beat blood pressure analysis demonstrated greater haemodynamic instability within the first 30 s after the command to stand up compared to measurements taken after achieving a stable standing position, underscoring the importance of continuous monitoring. Particularly in geriatric inpatients, who required between 3 and 90 s to reach a stable standing position, critical blood pressure fluctuations may go undetected with conventional measurement approaches.

The observed IOH prevalence (31.4%) in our study aligns with estimates from prior studies among older populations. A recent meta-analysis reported a pooled IOH prevalence of 29.0% among older adults assessed with continuous beat-to-beat blood pressure monitoring, with specific rates of 27.8% among community-dwelling older individuals and 35.2% in geriatric outpatients [4]. Similarly, the Irish Longitudinal Study on Ageing (TILDA) reported a prevalence of 34.7%, although this cohort included adults aged ≥ 50 years and employed continuous monitoring [9]. The high prevalence in our cohort may reflect the burden of frailty, multimorbidity, polypharmacy, autonomic dysfunction, and prolonged immobilisation that commonly characterise acutely hospitalised geriatric patients [22, 23]. These factors are well-recognised contributors to orthostatic blood pressure instability. Moreover, participants were assessed during acute illness, a state that may further exacerbate transient cardiovascular dysregulation during postural change [24]. Although the adverse outcomes associated with orthostatic dysregulation - including falls, syncope, and increased mortality - are well established [6, 25, 26], research specifically addressing IOH remains limited, particularly within inpatient populations [27]. Our findings highlight the need for future studies to investigate the clinical relevance of IOH in this population and to explore targeted management strategies.

A novel aspect of our study is the dual-timing assessment - measuring blood pressure both 15 s after the command to stand up and after the patient achieved a stable upright posture. This approach extends beyond previous studies, which have predominantly assessed orthostatic blood pressure changes at a single time point [4, 28], and likely improved detection sensitivity in hospitalised older adults, many of whom exhibit variable and prolonged standing transitions. Capturing haemodynamic responses across the full transition period may enable a more accurate and clinically meaningful identification of transient hypotensive episodes, which may otherwise be missed in conventional assessments. The median time to achieve standing up in our cohort was approximately 26 s, reflecting the functional impairments common in frail, multimorbid inpatients and underscoring the importance of adapting diagnostic procedures to real-world clinical conditions. Findings from TILDA support the impact of standing speed on orthostatic responses. O’Connor et al. reported that individuals who stood within 5 s experienced mean SBP and DBP drops of − 26.4 mmHg and − 16.4 mmHg, respectively, at 10 s after standing up. By contrast, those with slower transitions (20 s) showed smaller declines of − 15.7 mmHg (SBP) and − 7.7 mmHg (DBP) [9]. Similarly, De Bruïne et al. found that older adults with OH who stood up more slowly (mean 24 s) exhibited 5.9% and 7.1% smaller reductions in SBP and DBP, respectively, within the first 15 s after standing up, compared to individuals with normal standing speed (mean 11.5 s) [10]. Given the acute illness of our participants, it is plausible that their standing speed may improve after discharge, potentially revealing more pronounced IOH. Conversely, stabilisation of blood pressure regulation in recovery may reduce IOH incidence [8]. Longitudinal studies are warranted to investigate not only the trajectory of blood pressure responses but also changes in standing dynamics over time.

These findings also point to a methodological challenge: conventional tools, such as the active standing test following a 5- to 10 min supine rest, may fail to detect early blood pressure drops in patients requiring longer to reach an upright posture [14]. Assessing IOH only after stable standing risks missing critical haemodynamic changes during the transition phase [9, 29]. Our dual-timing protocol offers a more comprehensive and sensitive method for IOH detection in hospitalised older adults. Future studies should refine IOH assessment strategies to reflect real-world clinical realities, particularly in acute care settings and determine whether drops during the transition or after stable standing are more diagnostically and prognostically relevant. While our approach aims to reflect real-world postural transitions, we acknowledge that standing speed and muscular engagement may influence IOH detection. Passive tilt-table testing or controlled autonomic challenges during MRI protocols are recommended in future studies to better isolate autonomic cardiovascular dysfunction from mobility-related factors [30–32].

IOH is characterised by a blood pressure decrease within the first 15 s of standing, requiring high temporal resolution. Studies using continuous beat-to-beat blood pressure monitoring, such as finger photoplethysmography, consistently report higher IOH prevalence than those using intermittent oscillometric methods [4, 27, 33], emphasising the diagnostic advantage of continuous measurements. However, despite its diagnostic precision, the use of continuous monitoring in clinical practice remains limited. For example, in the RESORT study of geriatric rehabilitation inpatients, continuous beat-to-beat blood pressure monitoring with Finapres technology failed in nearly one-third of cases due to signal instability, insufficient peripheral perfusion, or positioning challenges despite repeated warming attempts [27]. Setup times often exceeded one hour, posing logistical barriers in routine clinical settings, particularly in frail, multimorbid older adults. However, procedural refinements may help mitigate some of these barriers. In our study, reliable measurements were generally achievable within 30 min, which may reflect the impact of several protocol adjustments, such as pre-warming the hand with moist heat, using appropriately sized cuffs for thin or arthritic fingers, minimising external pressure on the finger cuff during postural transitions, and providing standardised verbal instructions to reduce variability and motion artifacts [16]. While these adjustments appear to improve feasibility in the hospital setting, they do not eliminate the broader practical constraints, and validation in diverse clinical environments remains necessary. Future work should, therefore, focus on developing simplified, time-efficient, and patient-centered protocols - such as modified active stand tests or hybrid approaches - that maintain diagnostic sensitivity while improving clinical usability. The rapid evolution of wearables (e.g. sensor-equipped hearables capable of capturing movement, temperature or haemodynamic changes as well as pulse rate and blood pressure) may further enhance feasibility. Until such approaches are validated, establishing both the clinical relevance and operational practicality of IOH assessment will remain essential before it can be considered for broader integration into geriatric care or falls prevention strategies.

The observed trend towards lower compression stocking use among IOH patients further emphasises the clinical relevance of IOH detection. Patients with IOH were less likely to use compression stockings, potentially reflecting an underrecognition or underestimation of their orthostatic symptoms in clinical practice. Compression garments are frequently recommended in OH to mitigate symptoms via reduction of venous pooling. However, their use in older adults may be limited by practical barriers, including difficulties in application, discomfort, and low adherence, which can diminish their effectiveness in real-world settings [34, 35]. Nonetheless, future therapeutic strategies specifically targeting IOH are warranted.

Orthostatic intolerance symptoms were observed more frequently in the OH groups compared with the non-OH group. Although this difference did not reach statistical significance, the pattern may indicate a clinically relevant trend given the modest subgroup sizes and the inherent variability in symptom perception and reporting. Notably, more than one quarter of participants without OH also reported orthostatic symptoms, underscoring that such symptoms can arise in the absence of measurable blood pressure drops. This finding points towards alternative pathophysiological mechanisms, such as impaired cerebral autoregulation or autonomic dysregulation [36–38]. Participants with transient IOH reported numerically fewer symptoms after reaching a stable upright posture compared with the other groups. This observation is physiologically and methodologically plausible, as symptoms triggered by transient hypotension typically occur within the first seconds of standing up and may resolve rapidly. In our cohort, patients with transient IOH required a median of 26.6 s to achieve a stable posture, which may have dissociated the timing of symptom onset from the standardised assessment window and led to underreporting during the post-stabilisation phase [28]. The absence of significant differences in fall history between groups likely reflects the multifactorial nature of falls in geriatric inpatients, where factors such as polypharmacy, cognitive impairment, and impaired mobility often outweigh the contribution of transient orthostatic events [39].

A non-significant trend towards prolonged hospitalisation was observed in patients with IOH, which should be interpreted with caution. While speculative, such a trend could reflect underlying physiological vulnerabilities - such as subclinical autonomic dysfunction or impaired cerebral autoregulation - that may delay recovery. However given the homogeneity of our sample (frail, multimorbid, and acutely ill) and the structural requirements of the national framework of acute Geriatric care in Germany, variability in length of stay was likely limited, thereby reducing statistical power to detect clinically meaningful differences. Overall, our findings do not support a robust association between IOH and hospital stay, but remain in line with prior work suggesting that subtle autonomic dysfunction or impaired cerebral autoregulation contributes to delayed recovery in this population [36, 40]. Van Twist et al. similarly proposed that adverse outcomes are more closely related to impaired haemodynamic recovery after IOH rather than IOH itself [41], supporting the notion that IOH may reflect underlying physiological vulnerability. While direct evidence remains limited, these observations are consistent with previous studies linking orthostatic hypotension to prolonged hospitalisation in older adults [5, 6, 22]. Potential contributing factors include cerebral hypoperfusion, increased fall risk, delayed mobilisation, and complex care needs. Although prior studies have associated IOH with frailty and falls [6, 28], its impact on hospital outcomes remains underexplored. Given its potential clinical relevance, further research in more heterogeneous cohorts, across centres and healthcare systems with different structural conditions, may provide further insights into these findings.

A key strength of this study is the use of continuous beat-to-beat blood pressure monitoring, which allowed for detailed temporal analysis of haemodynamic changes during postural transitions and facilitated accurate identification of IOH. Conducted in a real-world acute care setting, the study included a clearly defined cohort of geriatric inpatients, enhancing its clinical relevance and applicability to similar hospital populations. The inclusion of patients with a broad spectrum of comorbidities, functional impairments, and medication regimens reflects the heterogeneity typical of geriatric care, further supporting the generalisability of our findings. In addition, the dual-timing assessment approach - capturing blood pressure changes both shortly after the command to stand up and upon achieving upright posture - provided a more nuanced understanding of orthostatic blood pressure dynamics.

However, several limitations must be acknowledged. The study was conducted at a single centre with a modest sample size, potentially limiting statistical power to detect subtle associations. Orthostatic assessments were performed at variable times of day and without full control over factors such as recent food intake or medication timing, which may have introduced confounding. The observational design precludes causal inference, and larger prospective studies are needed to clarify the potential impact of IOH management on clinical outcomes. As functional status and clinical characteristics did not differ between subgroups, more detailed assessment e.g. timed up and go test or short physical performance battery) may reveal subtle impairments not captured by the Barthel Index. However, such assessments can be challenging to implement in acutely ill geriatric patients. Future studies would benefit from incorporating these measures, reporting not only their outcomes but also their feasibility and the diagnostic value of individual subscores (e.g. from the SPPB). Although continuous blood pressure monitoring was generally feasible, signal acquisition was occasionally compromised, particularly in patients with poor peripheral perfusion or movement artefacts. Further efforts are needed to enhance the reliability and ease of use of such monitoring in acutely ill older adults.

## Conclusion

The study highlights IOH as a prevalent condition in hospitalised geriatric patients, characterised by haemodynamic instability during postural transition. The findings emphasise the limitations of relying exclusively on intermittent measurements as usually applied, which may overlook transient hypotensive episodes occurring in the early phase after standing up. Incorporating continuous beat-to-beat monitoring and adapting diagnostic protocols to the physiological characteristics of frail older adults may enhance detection and clinical decision-making. Although IOH showed no statistically significant associations with clinical outcomes in our sample, it may, nevertheless, indicate underlying physiological vulnerability in frail, multimorbid individuals. Given the practical challenges of continuous monitoring in routine care, broad implementation is unlikely to be feasible at present. However, targeted assessment in selected high-risk groups - such as patients with recurrent falls, syncope, delayed mobilisation, or suspected autonomic dysfunction - may be of clinical value. Future research should aim to optimise the feasibility of diagnostic protocols and to determine whether targeted management of IOH can meaningfully influence recovery trajectories or long-term outcomes in this population.

## Data Availability

The dataset used and analysed during the current study are available from the corresponding author upon reasonable request.
